# A ‘Dilute and Shoot’ Liquid Chromatography-Mass Spectrometry Method for Multiclass Drug Analysis in Pre-Cut Dried Blood Spots

**DOI:** 10.3390/ijerph18063068

**Published:** 2021-03-16

**Authors:** Lucia Mainero Rocca, Nunziata L’Episcopo, Andrea Gordiani, Matteo Vitali, Alessandro Staderini

**Affiliations:** 1 Chemical Agents Laboratory, Department of Occupational and Environmental Medicine, Epidemiology and Hygiene, Italian Workers’ Compensation Authority (INAIL)—, Via Fontana Candida 1, Monte Porzio Catone, 00078 Rome, Italy; n.lepiscopo@inail.it (N.L.); a.gordiani@inail.it (A.G.); staderini.1644817@studenti.uniroma1.it (A.S.); 2 Department of Public Health and Infectious Diseases, University of Rome La Sapienza, P.le Aldo Moro, 5, 00185 Rome, Italy; matteo.vitali@uniroma1.it

**Keywords:** sleep inducers, ototoxic drugs, pre-cut dried blood spots, whole blood analysis, dilute and shoot, UPLC-MS/MS, work safety

## Abstract

Drugs able to affect the auditory and nervous systems and consumed by workers to treatdifferent pathologies can represent a possible source of risk in the work environment. All the target compounds involved in the presented project show ototoxic and/or narcoleptic side effects and, for these reasons, occupational safety organizations have recognized them as potential causes of work injuries. A multiclass method for the analysis of 15 drugs among the most widespread worldwide (belonging to nine different classes including antihistamines, beta-blockers, antidepressants, Z-drugs and opioids), was developed and validated. This study describes a rapid, sensitive and effective method to analyse these substances in whole blood using tailored pre-cut dried blood spots. Detection was achieved with a triple quadrupole mass spectrometer after an easy and simple ‘dilute and shoot’ solubilisation followed by an UPLC separation. All the issues linked to the use of the dried blood spots and whole blood, such as haematocrit variability, volumetric evaluation and sample carrier choice were carefully studied and managed during method development. From the validation study results it emerged that this approach can be deemed successful thanks to its few pg µL^−1^ LOQs, good linear intervals, absolute recoveries of no less than 75%, an almost negligible matrix effect and accuracy and precision in line with the European and American guidelines for validation. All the obtained goals have been specifically pursued in order to encourage method diffusion as a primary prevention intervention, even in small private workplaces.

## 1. Introduction

Occupational safety requires a 360-degree study of the factors that can constitute a risk for the figures involved. Among all the possible sources of danger, risks arising from chemicals is certainly one of the most complex to understand and to manage, whose assessment is usually devoted to the evaluation of types and concentrations of the substances to which workers are potentially exposed.

However, this classical approach has a ‘blind spot’ since workers, like everyone else, are exposed to chemicals in their private life, and, like everyone else, they take drugs whose side effects can pose a safety risk on several levels.

The presented project was designed to cover a part of this often-neglected aspect. All the chosen analytes, in fact, show side effects focused on the hearing and nervous system. They belong to the antihistamines, antidepressants (serotonin-norepinephrine and selective serotonin reuptake inhibitors), antihypertensive, beta-blockers, anxiolytics (benzodiazepines), opioids and Z-drugs classes.

From different studies conducted by the Organization for Economic Co-operation and Development (OECD) in 2016 and by the Italian Medicines Agency in 2018, these drugs appeared to be the most widespread in markets. Specifically, anti-hypertensive and lipid-lowering drugs are among the leading therapeutic classes (even if they saw a decline in the last few years) while analgesics (including opioids) and drugs used to treat psychiatric disorders follow at a short distance [1,2,3].

The selected drugs have a narcoleptic effect and many of them affect the middle and inner ear too, causing dizziness or tinnitus up to hearing loss [4,5]. Benzodiazepines, hypnotics, opioids and beta-blockers are cause of sensorineural hearing loss due to dysfunction of the cochlea; moreover, even a single dose of these classes of drugs can significantly impair body balance leading to dangerous falls [6,7].

The occupational health and safety literature reveals that the use of these sleep inducers may negatively affect the performance of safety-sensitive work tasks such as driving or operating machinery, and consequently increasing workers’ compensation costs [8,9]. Our Institute, through its studies, has highlighted that many workplace accidents can be linked to ear problems and/or lack of attention [10,11]. The collected data show that at least 10% of work accidents are attributable to auditory and psychic-behavioral pathologies. Although evaluation of these molecules’ consumption in workers is not mandatory (except for specific drugs of abuse), their use is increasingly becoming a parameter that merits further investigation [12]. Therefore, from the perspective of ensuring 360° worker protection, our goal was to develop and validate a fast and reliable method for the analysis of these drugs in whole blood.

With this purpose, the time-consuming extraction step was bypassed developing a lighter procedure, the so-called ‘dilute and shoot’-LC-MS (DS-LC-MS) method. Generally applied to urine samples and often used in anti-doping controls [13,14,15], we decided to transfer it to dried blood spots (DBS) with the same intent, i.e., the development of a fast and cost-effective procedure.

DBS have gained an important role among the whole blood sampling techniques, thanks to their extreme simplicity of collection, transportation and storage. Blood, in fact, is taken through a small puncture on the finger or the heel (or on tail for animals), placed on a support and allowed to dry. After that, sample can be sent as a simple letter avoiding any need for following bio-hazard or cold chain procedures.

DBS has been used for screening of diseases in new-born since 1963 [16,17,18] but, recently, they have attracted attention in different fields such as therapeutic drug monitoring [19,20], home sample collection, pharmacokinetic studies and drug resistance [21,22], anti-doping and forensic controls [23,24].The DBS technique offers numerous advantages over the traditional venous sampling that can be performed only in equipped health-care facilities by specifically trained personnel, and that represents a burden for the patient. Small sample volume, increased analyte stability (due to lower enzymatic activity in dried blood), safety and low-cost shipment are among blood spot analysis’ most evident benefits.

Despite the mentioned ‘pluses’, this sampling technique has still some drawbacks that must be addressed. Two of the most preponderant of these are blood viscosity and haematocrit (HCT) that have a non-negligible influence on quantitation results as well as the nature of the support material from which analytes have to be extracted [25]. Another source of result unevenness is due to the possibility that capillary blood may differ in terms of analyte composition and matrix effects from venous blood. When this occurs, DBS outcomes may be very different from traditional venepuncture ones [26,27]. No specific studies exist on the drugs selected for this study, but a vast literature concerning the correlation between capillary and venous concentrations is available [28,29,30,31,32]. The majority of these studies suggest a venous/capillary concentration ratio near 1.00 (with small fluctuations) and with closely correlated concentrations [33,34,35].

To overcome the variability arising from blood viscosity, especially the haematocrit impact, spotting technique and conditions, two groups of researchers modified the traditional blood spots technique by developing the pre-cut dried blood spot (PCDBS) method [36,37]. For this purpose, the support chosen for the DBS was cut before being loaded with blood, thus, the whole droplet is sampled and not only the part selected by the cutter.

The aim of this project was to apply this elegant and simple sampling procedure to the analysis of different classes of ototoxic and narcoleptic drugs in whole blood. However, the use of small volume of sample is at the same time an advantage and a weakness whose only solution would be the adoption of very sensitive and powerful instrumentation. This requirement also dictated by the low blood concentrations of the selected drugs at therapeutic doses [38,39,40,41]. These considerations led us to the use of an UPLC system for the analyte separation and a tandem mass spectrometer for the detection.

Among the selected analytes, we noticed that synthetic opioids and benzodiazepines are widely studied and evaluated in biological and/or environmental matrices with many purposes. In contrast, substances such as trazodone, some antihypertensive and antihistamines are almost neglected by the scientific community but not less worrying ij the context of interest. To the best of our knowledge, at present there are no other reports on the analysis of all these ototoxic and narcoleptic compounds simultaneously with a simple ‘dilute and shoot’ procedure applied to whole blood on DBS.

## 2. Materials and Methods

### 2.1. Chemicals

First generation H_1_-antihistamines (diphenhydramine hydrochloride, cyproheptadine hydrochloride, triprolidine hydrochloride); second generation H_1_-antihistamines (cetirizine hydrochloride, ebastine); a selective serotonin reuptake inhibitor (SSRI) class antidepressant (escitalopram oxalate (*S*-(+)-citalopram oxalate)), a serotonin antagonist and reuptake inhibitor (SARI) class antidepressant trazodone hydrochloride, a serotonin-norepinephrine reuptake inhibitor (SNRI) class antidepressant venlafaxine hydrochloride, opioids (fentanyl and tramadol hydrochloride, also a SNRI class compound); benzodiazepines (alprazolam, bromazepam, delorazepam); a beta-blocker (atenolol) and the Z-drug zolpidem with purities ≥98% were purchased from Sigma-Aldrich (Merck Life Science, Milan, Italy). The internal standards (ISs) used were atenolol-d_7_ and diphenhydramine-d_3_ from Sigma-Aldrich; cyproheptadine-d_3_, cetirizine-d_8_ and tramadol-d_6_ purchased from Alsachim (Shimatsu Corporation, Graffenstaden, France) and ebastine-d_5_ from Clearsynth (Clearsynth Labs, Villeurbanne, France).

Individual stock solutions of all the analytes were prepared at 1 mg mL^−1^ in methanol and stored at −18 °C for at least 6 months. Working standard solutions and mixtures were obtained by dilution of the above ones to the appropriate concentrations.

Acetonitrile and methanol, both MS grade, were from Carlo Erba (Milan, Italy). Formic acid puriss. p.a. was purchased from Sigma-Aldrich. Deionized water was obtained from a Milli-Q Plus system (Millipore, Bedford, MA, USA).

### 2.2. Dried Blood Spot Sampling

**Figure 1:**
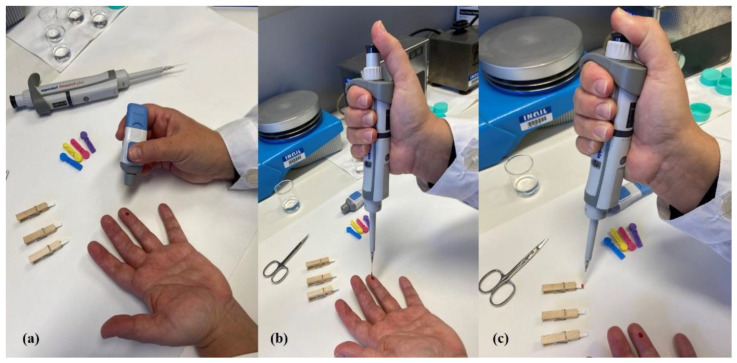
Three pictures that summarize the whole blood sampling procedure. Blood was taken with a lancing device (Panel (**a**)) and 2 µL were collected with a pipette (Panel (**b**)); finally, it was loaded on the paper support (Panel (**c**)). One of the authors has volunteered to carry out the sampling on herself in order to show how easy it is to carry out the procedure alone if necessary.

Whole blood samples were collected from the ring fingers of volunteers by piercing it with a lancing device; the spilled blood is drawn with a pipette (2 µL) and then loaded onto a pre-cut cellulose filter paper (15 × 4 mm; weight 67 g m^−2^, thickness 0.13 mm) from Biosigma s.r.l. (Venice, Italy). Figure 1 visually summarizes these first steps.

**Figure 2:**
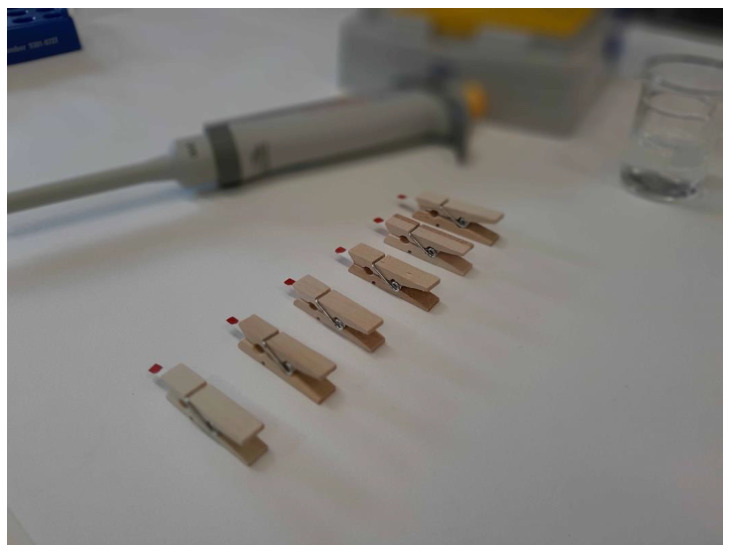
DBS homemade support; the wooden clothespin allows the blood deposited on the paper to dry uniformly and avoid any contact with the bench. Samples can be cut and placed in Eppendorf vials simply by using the clips as a handle.

Samples were allowed to dry for 10 min raised off the bench by means of a homemade support (see Figure 2). During first stage of method development and validation venous blood was used; it was stored inside under-vacuum heparin tubes (BD Vacutainer^®^, BD s.r.l., Milan, Italy) and kept for less than 2 weeks refrigerated at −18 °C.

### 2.3. Dilute and Shoot Procedure

Filter paper, loaded with blood, was cut from the homemade support in order to obtain a sample as small as possible and placed into a 2 mL Eppendorf safe lock tube (Eppendorf s.r.l., Milan, Italy). Fifty µL of distilled water was added and sonicated for 5 min; then 50 µL of acetonitrile, containing a suitable amount of ISs solution, was added and the mixture was again sonicated for 5 min. The obtained mix was centrifuged on an Eppendorf 5430R centrifuge (Eppendorf s.r.l.) for 8 min at 8000 rpm and 20 °C. This solution, deprived of any corpuscles, was placed in a vial and a volume of 2 µL was directly injected in the UPLC system without further treatment.

### 2.4. Liquid Chromatography and Mass Spectrometry

Separation of the 15 analytes was obtained with a gradient elution on a Kinetex Biphenyl column (100 × 2.1 mm and 2.6 µm particle size, Phenomenex Inc., Torrance, CA, USA). The mobile phases used were acetonitrile (A) and water (B) both containing HCOOH 5 mM at the flow rate of 0.5 mL min^−1^. The flow profile was as follows: 0–4 min from 10% A to 40% A; 4–6 min from 40% A to 100% A; 6–8 min 100% A.

Chromatographic runs were performed with an Acquity^TM^ ultra-performance liquid chromatography (autosampler and binary solvent management) system from Waters (Milan, Italy). Mass spectrometric detection was accomplished on an API 4000 QTRAP^®^ hybrid triple quadrupole/ion trap mass spectrometer (AB Sciex, Concord, ON, Canada) equipped with a Turbo V ion spray source.

Two fragments for each analyte were selected in order to work in multiple reaction monitoring (MRM) mode. All the parameters relating to signal transmission inside the mass spectrometer, from the source to the electron multiplier, were carefully optimized infusing each single standard solution at a concentration ranging between 0.01 and 1 mg L^−1^ by a syringe pump (flow rate 10 μL min^−1^). Nitrogen was used as curtain, nebulizer, drying and collision gas (30, 60, 60 and ‘medium’, respectively, manufacturer’s units); the drying gas temperature was set at 500 °C. All the analytes were detected in positive ionization mode. Instrument calibration was periodically checked for each mass analyzer (Q1 and Q3) by the infusion of a solution of polypropylene glycol at 10 μL min^−1^. Unit mass resolution was established and retained in each mass resolving quadrupoles by maintaining a full width at half-maximum of approximately 0.7 ± 0.1 Da. Data were acquired and processed with the Analyst 1.5.1 software (AB Sciex). Names, structures, retention times and optimized essential mass spectrometric parameters of the target compounds are shown in Table 1.

### 2.5. Method Validation

DBS is a peculiar sampling technique and, therefore, needs specific protocols to ensure method performance. In this paper, validation was performed following the suggestions of Capiau/Veenhof et al. [42], the European Medicines Agency and the U.S. Food and Drug Administration guidelines [43,44].

It was unrealistic to use blood from a finger prick for validation method purposes and for this reason, all the samples used were obtained from venipunctures. Blank samples, treated with heparin, were divided into 2 mL portions and stored at −18 °C when unused. For each step of the validation protocol, a suitable amount (in the minimum volume) of analyte standard solutions were added to a 100 µL of sample and left to equilibrate at room temperature for 30 min. The suitable amount of the internal standards solution was added during dilution step and before the centrifugation.

Limits of detection and quantitation were calculated on the less intense MRM transitions among the two selected for each analyte (qualifier) while all the other parameters were determined on the most intense ones (quantifier). This approach guarantees three identification points which, and in combination with the retention times, allow an unequivocal identification of the analytes. Limits of Detection (LODs) and Limits of Quantitation (LOQs) are experimentally estimated by gradually decreasing the spiked analyte concentration until it reaches a signal equivalent to three (or 10, respectively) times the background noise.

Selectivity was verified on all the standard solutions and on the blank samples used for the development protocol. Each standard and internal standard was injected in the LC-MS system at 10 × LOQs and the absence of reciprocal interference was verified. Twenty blank samples of whole blood were analyzed to check the absence of interferent peaks in the retention time window of each target analyte.

The linear dynamic range for each analyte was evaluated in whole blood for at least one order of magnitude starting from LOQ values to 10 × LOQs. For this purpose, a set of five samples spiked at the end of the dilution procedure with both standards and internal standards solutions was injected and a linear regression calculation was used to construct the calibration curve. ISs concentrations were at the same concentration of the homologs at the 5 × LOQs values. In this occasion matrix effect was carefully checked for each analyte calculating the decrement of the angular coefficients of two calibration curves: one on blood samples and one on standard solutions in methanol.

Absolute recoveries and precision were measured on three different levels of concentration, six samples for each level, (low = LOQ; medium = 2.5 × LOQ and high = 5 × LOQ) and precision was expressed as relative standard deviation. Absolute recoveries were determined by comparing the chromatographic peak areas of the sample subjected to the complete dilution procedure with the ones obtained from the extraction of a blank sample spiked at the end of the procedure. Intra-day precision was estimated analyzing six spiked samples for each level; the same set of analysis were repeated on three different days to assess the inter-day precision.

Accuracy was expressed as a percentage of the real value of the measurand; therefore, the three validation levels (six samples each) were analyzed against the calibration curve to compare the obtained and the real values.

Stability protocol was established in order to understand how long the DBS can be stored without having a significant drop in the analytes concentration. 20 samples (10 Low and 10 High) were used for stability protocol. Samples were stored at −18 °C in plastic boxes and the check points were 0, 7, 15, 30 and 90 days. All the calculations were performed using analyte peak area vs IS peak area ratios.

## 3. Results and Discussion

### 3.1. Haematocrit (HCT) Management

Haematocrit is the most challenging issue in a DBS quantification method development [45]. This parameter represents the ratio between the red blood cell volume and the total blood volume, generally expressed as a percentage. In a certain way, HCT can be associated to blood density and viscosity, as mentioned in the Introduction section. For this reason, it is associated to blood drop size and, therefore, to spot size and, as a consequence, to the quantitation results [46]. It means that a high HCT level corresponds to a smaller spot surface and to a higher analyte concentration in the DBS (and the other way round) [47].

The presented method adopted two approaches in order to reduce and control the preponderant influence of HCT, thus allowing to perform a quantitative analysis: the use of a re-cut support and a defined blood volume.

A PCDBS procedure means that the entire blood volume undergoes the analysis protocol and therefore biases are not ascribable to hematocrit value [36,37]. This technique allows an accurate and valid determination regardless of the HCT level and blood viscosity. Moreover, by using a pre-cut carrier it is possible to avoid any carryover due to the use of a manual or semi-automated puncher.

Since the goal of the project was a quantitative analysis, a measured volume of blood is applied on the pre-cut filter paper by using a calibrated micropipette (2 µL), collecting sample directly from the ring finger. However, the pipette tip should not touch the DBS surface in order to avoid paper damage. Multiple layers should be avoided too, since they could lead to a bigger spot and to an overload. The practice of sampling with a pipette or a capillary tube is not new [48] and, in our opinion, not too difficult to practice also at home [49]. Although the developed procedure is designed for worker investigations, in fact, it can be easily used in home withdrawals, as illustrated in Figure 1.

Another source of bias related to HCT is due to intra- and inter-individual variability and factors such as the age, sex and health of the donor [50]. The mean value is generally among 40–54% and 36–46% for men and women, respectively. However, for newborns, for instance, the values are between 53–69% like for people living at high altitudes, while microcythemic subjects, even if not anemic, have a lower HCT level (smaller volume of red blood cells).

The developed method was tested on different subjects and different types of blood were analyzed in order to understand if individual variability could affect the results. The samples were a small group of women and men of different ages and a young microcythemic subject.

Differences between the samples were immediately clear from a preliminary examination as, for example, differences in color and opacity. However, the simple procedure optimized for the analysis of the 15 substances seemed to be unaffected by these variables: and no differences in matrix effect nor in extraction efficiency were recorded

### 3.2. Pre-Cut Spots, Support Materials and Dilution

Taking into account the authors’ previous experience with direct analysis methods, support optimization was conducted choosing different suitable materials starting from the hydrophobic ones. Substances were evaluated and compared in terms of recovery percentage, blood diffusion and handling.

Knowing the chemical-physical characteristics of both matrix and analytes, the extraction solvents under consideration were reduced to three: water, methanol and acetonitrile. All the supports were treated with a combination of them in order to confront extraction efficiencies: H_2_O, H_2_O:CH_3_OH (50:50) and H_2_O:CH_3_CN (50:50). Further investigations were carried out for the most promising combinations.

A PTFE filter (Merck Omnipore, 0.45 µm, 47 mm), acetate film and wax paper were the first to be tested. The last two showed good results during spot deposition, associated with recoveries between 41% and 95% (except for trazodone, fentanyl, cetirizine and escitalopram that had lower values), but they were too lightweight and flexible. On these materials, the dry drop breaks and detaches from the support since it is not equally flexible becoming difficult to handle and to transport. The highly hydrophobic PTFE surface showed an anomalous behaviour: the 2 µL blood drops stayed as perfect spheres on its surface even when dry. 

**Figure 3:**
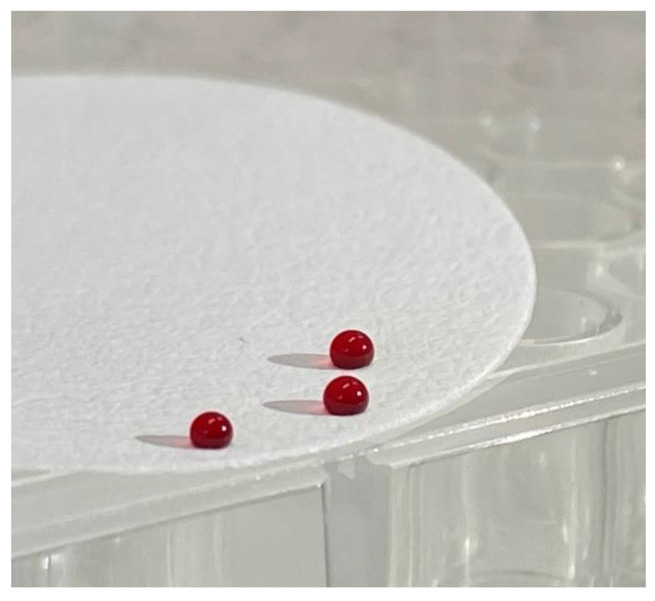
A 2 µL blood drop deposed on a PTFE filter; the drop remained spherical even after drying.

Dry spheres (shown in Figure 3) remain firmly stuck to the surface allowing a good handling, but the 3D structure compromises a simple storage and shipment. Moreover, analytes showed a greater affinity for Teflon rather than for the dilution solvents and this led to low recoveries.

**Figure 4:**
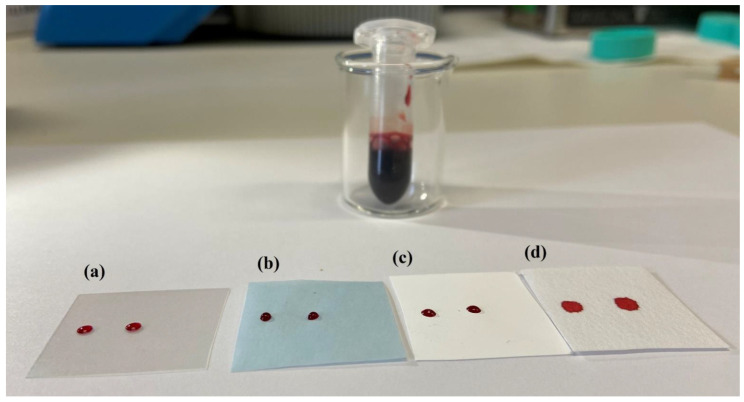
Comparison of the different behaviors of the same volume of whole blood (2 µL) on different surfaces. Substrate (**a**,**b**) are acetate film and wax paper, respectively; (**c**,**d**) are cellulose mixed ester and bench paper respectively.

Hydrophilic supports, on the contrary, completely absorbed the drops spreading it on a large surface. Different celluloses (types, pores, thickness, absorbing power) were evaluated. The diverse behavior, showed by whole blood on each substrate, is illustrated in Figure 4.

Filter paper showed the higher percentage of recoveries for all the analytes, especially when processed with water and acetonitrile (R ≥ 60% except for atenolol and bromazepam). When the two solvents were added in sequence instead of simultaneously, recoveries increased to R ≥ 75% for all the analytes, as shown in Table 2.

The spreading issue was solved pre-cutting the filter paper in small stripes with a width of 4 mm (PCDBS). Although the spot size is not particularly incisive on the analysis, the pre-cut support was made as smaller as possible. The goal was both to contain the drop and to minimize the elution volume and, therefore, the dilution factor. The use of an ultra-sound device after each solvent addition improved the dry blood wettability and helped to dilute the spot.

In order to prevent contact between humid samples and every surface, we developed a homemade wooden device using a common clothespin. As can be seen in Figure 2, the paper strips were firmly held between the two prongs and then spiked with blood. This clothespin also helps to handle samples once they are dry, avoiding any risk of cross contamination.

### 3.3. Dilute and Shoot and Chromatographic Separation

The final solubilisation procedure resulted very similar to the so called ‘dilute and shoot’ technique, generally applied to urine analysis [51,52]. Therefore, it was decided to deepen our study and evaluate its performance when applied to a different matrix such as whole blood.

This technique is really simple, fast and reliable and, in fact, it is common among doping and forensic analyses. However, in the recent years, due to a trend that led to a more simplified sample pre-treatment, DS is finding new applications [53,54,55,56]; nevertheless, it was rarely applied to whole blood or to DBS and only to human and animal plasma [57]. Clearly, the achievement of good results in terms of LODs and reproducibility is strongly dependent on the affinity of the analytes for the ionization technique.

Chemical-physical properties of the target compounds favoured the application of this fast sample treatment showing a great ionization efficiency. For instance, the easy ionisable basic nitrogen groups on the hypnotic drugs had a high response to ESI voltage and that was clear since MS/MS parameters optimization step in which a really high signal was registered even during the initial Q1 scan. Bromazepam had a different behaviour, maybe due to the presence of the halogen. In general, all the analytes had a good ability to coordinate a proton and that meant higher performance of the DS-LC-MSMS analysis. With a blood volume of only 2 µL deposited on the support, the final dilution factor was 1:50, nevertheless good method limits were achieved as shown by the LOD values (Table 3).

A brief centrifugation step was added prior to the chromatographic separation in order to prevent any sediment (from blood or paper) from entering the UPLC system and possibly blocking it. Generally, only small amount of particles remains at the bottom of the Eppendorf vials after this part of the procedure. At the same time, both the centrifugation and the dilution factor help to avoid instrument contamination.

A constant monitoring of potential carry-over or cross contamination was scheduled by carrying out two solvent injections after the most concentrated points of the calibration curves along all the analytical session. No memory or carry-over effects were observed. Moreover, despite the simplicity of the procedure, the absolute recoveries proved to be quantitative.

The 15 analytes selected for this project cover a vast range of chemical and physical properties and, therefore, chromatographic separation was complicated by many factors. Thus, several stationary phases have been tested in order to reach an acceptable separation and a good peak shape.

C_18_s were the first to be evaluated, due to their great versatility and, among them, different selectivity and polarity were tested maintaining the same mobile phases. Unexpectedly, neither the silica nor the hybrid particles showed acceptable results.

Waters Acquity UPLC^®^ HSS T3 (100 × 2.1 mm, 1.8 µm), BEH C_18_ (50 × 2.1 mm, 1.7 µm) CSH™ C_18_ (100 × 2.1 mm, 1.7 µm) with silica-based end-capped particles showed large peaks for venlafaxine, diphenhydramine and particularly for triprolidine. The low-level surface charge of the CSH, in addition, split atenolol into two peaks at the beginning of the chromatographic run.

**Figure 5:**
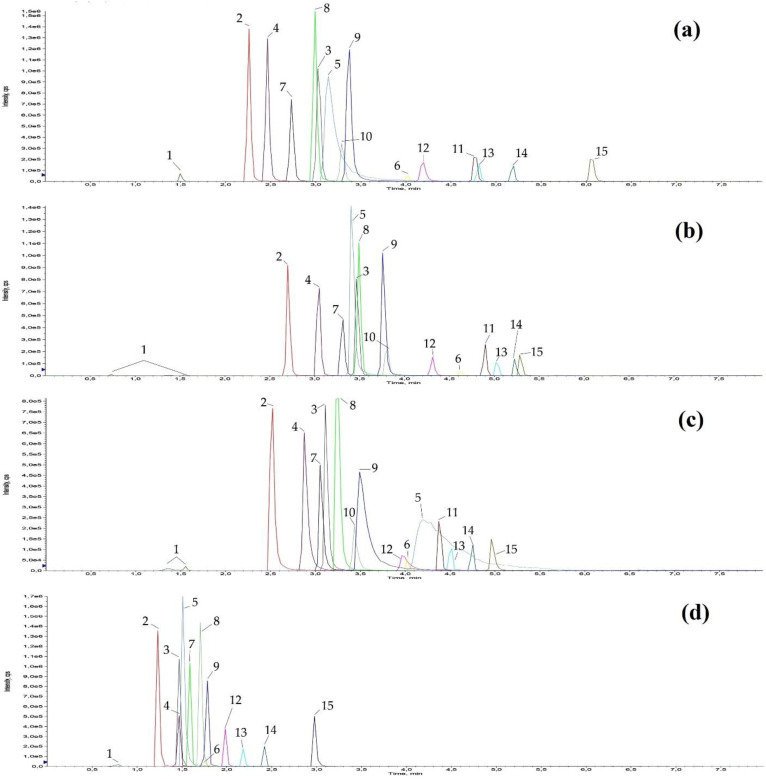
Chromatographic comparison of some of the C18s tested for the analytes separation (2 ng injected). Panel (**a**): Acquity UPLC^®^ HSS T3; Panel (**b**): CSH™ C_18_; Panel (**c**):Kinetex^®^ EVO C_18_; Panel (**d**): Kinetex^®^ C_18_. For a simpler visualization, the peaks have been indicated with numbers to which the following analytes correspond: 1 atenolol, 2 tramadol, 3 venlafaxine, 4 zolpidem, 5 triprolidine, 6 bromazepam, 7 trazodone, 8 fentanyl, 9 diphenhydramine, 10 escitalopram, 11 cetirizine, 12 cyproheptadine, 13 alprazolam, 14 delorazepam, 15 ebastine.

Better results were obtained switching to an organo-silica ethane cross-linking core-shell particle column (Kinetex^®^ C_18_, 100 × 2.1 mm, 1.7 µm, from Phenomenex), while the Kinetex^®^ EVO C_18_ (100 × 2.1 mm, 1.7 µm) showed a similar behaviour to the Waters columns. A visual summary of these results is shown in Figure 5.

Moving to stationary phases with a combination of hydrophobic, aromatic, and polar selectivity such as phenylhexyl, pentafluorophenyl and biphenyl, allowed us to achieve better resolution and peak symmetry. The best compromise in terms of chromatographic resolution, peaks shape and signal-to-noise (S/N) ratios was obtained with the Kinetex^®^ biphenyl column (100 × 2.1 mm, 2.6 µm) in combination with H_2_O and CH_3_CN as mobile phases.

The mechanism that most likely underlies this improvement is the dipole moment correlation; a smaller contribution is probably given by shape selectivity and polarizability [58]. The aromatic ring of the biphenyl moiety has a net negative charge on both sides of the ring itself, but it is not clear if the interaction with analytes is due to π-π, charge transfer, or polarity interfering with stationary phase solvation.

**Figure 6:**
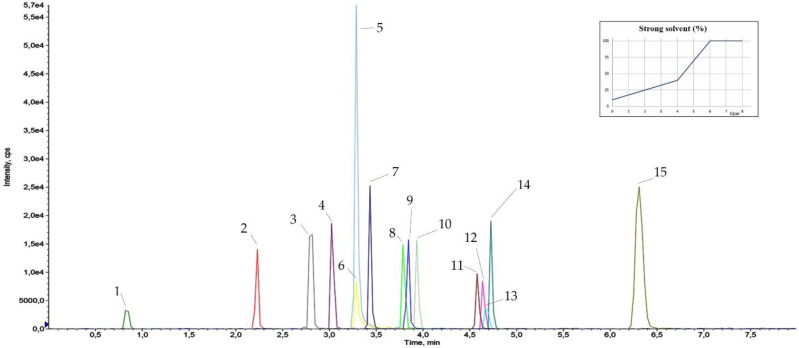
Chromatogram (10 pg injected) obtained with the final conditions (see paragraph ‘Liquid chromatography and mass spectrometry’) the scheme showed the strong solvent (CH_3_CN) percentage trend during the run. Peaks names: 1 atenolol, 2 tramadol, 3 venlafaxine, 4 zolpidem, 5 triprolidine, 6 bromazepam, 7 trazodone, 8 fentanyl, 9 diphenhydramine, 10 escitalopram, 11 cetirizine, 12 cyproheptadine, 13 alprazolam, 14 delorazepam, 15 ebastine.

In order to obtain the highest performance from the Biphenyl column, mobile phases’ strength, pH and concentration of organic modifier have been finely tuned. The result of the large amount of tests carried out to optimize gradient, flow rate and formic acid concentration is shown by the final chromatogram, represented in Figure 6.

### 3.4. Results of Method Validation

Selectivity studies were carried out both on matrix and standard solutions; greater attention was paid to the internal standard solutions and to the potential presence of the non-isotopic homologs. The absence of intrusive signals in any MRM chromatogram highlighted that interference from the whole blood or from standard solutions themselves were totally absent.

Since the method is multi-class, limit of detection and of quantitation were empirically evaluated for every analyte on blank whole blood spiked samples. Results were noticeably low, demonstrating a good method ‘sensitivity’ despite the 1:50 sample dilution. LODs, in fact, were all below 1 pg µL^−1^ except for bromazepam and ebastine (4.9 and 1.4 pg µL^−1^, respectively); LODs and LOQs values are reported in Table 3.

Linear regression calculation showed that ion signals were linearly correlated over the selected concentration range with a coefficient of determination (R^2^) in matrix not less than 0.998.

The developed DS procedure and a suitable gradient elution have proven to be particularly effective in reducing matrix effect on the MS signal. The comparison between solvent and matrix calibration curves displayed that the decrease in method sensitivity was negligible for most of the analytes (less than 20%) except for cyproheptadine that showed a higher suppression (29%). All the matrix effect values are reported in Table 3.

A possible side effect, due to the lack of an initial clean-up, could be a scarce reproducibility of the retention times. For this reason, a set of 20 spiked samples (5 × LOQs) were analyzed, in order to test a potential variability of each analyte’s t_r_. The obtained values in term of relative standard deviations were less than 2.5%.

Absolute recoveries (R%) were calculated in order to evaluate the real amount that DS procedure was able to extract from filter paper used as sample carrier. Outcomes showed that all the drugs were recovered quantitatively and that the two-step procedure is effective, leading to R% ≥ 75% (see Table 2).

For method precision and accuracy evaluation, we decide to analyse three levels instead of four as recommended by EMA and FDA [42,43,44]. The two organizations, actually, suggest the use of a lower limit of quantitation (five times the S/N ratio) as one of the validation levels. Nevertheless, it is our opinion that a more precautionary position of the lowest limit at which a quantification is effective is a better choice; therefore, we preferred not to drop below the LOQ (10 times the S/N). The use of LOQ-multiples of LOQ interval is a well-established and accepted practice in validation of trace analysis methods. For these reasons, we used LOQs as the lowest level and 5 × LOQ as the highest (medium 2.5 × LOQ).

Intra-day precision, expressed as relative standard deviation (RSD %), was well below the prescribed 15%; in fact, for all the validation levels values resulted between 2.5 and 10.2%. Inter-day precision likewise met the acceptance criteria, showing all values between 4.3 and 14.9%. Method accuracy was determined by comparing the measured concentrations with the calibration curves and expressed as trueness. All the results obtained from validation process are shown in Table 2.

### 3.5. DBS Stability

Due to a reduced enzymatic activity, DBS are less prone to analyte concentration fading. Nonetheless, blood samples exhibit physiological aging and therefore a protocol has been adopted to evaluate drugs blood concentration as a function of time and storage condition. Less than 15% decrease from the nominal concentration was considered acceptable, as recommended by Capiau/Veenhof et al., EMA and FDA [42,43,44].

Tests were scheduled over a period of 90 days; the T_0_ sample was left to dry at high temperature and humidity (30 °C and 56%, respectively) for two hours. The goal was to simulate a worst-case real condition for sampling: many workers and a humid and hot workplace. For the remaining check points (7, 15, 30 and 90 days) samples were stored at −18 °C.

**Figure 7:**
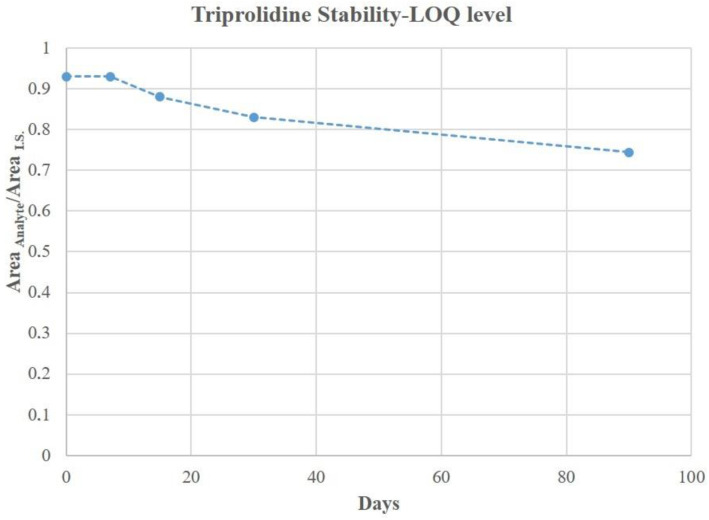
Triprolidine concentration trend over a period of 90 days chosen for stability control (LOQ level). On the y-axis is reported the analyte area vs IS area ratios.

Results showed that filter paper combined wit cold storage allowed us to maintain almost unaltered analyte concentrations for up to 30 days. After this period a slow decrement emerged for all the selected drugs. The triprolidine concentration trend for the lowest concentration at the stability checkpoints is shown as an example in Figure 7.

## 4. Conclusions

There are several statistics indicating that the use of ototoxic and narcoleptic drugs poses a risk in the workplace. Current legislation does not consider it necessary to evaluate these substances in biological fluids in order to control their use and to protect workers’ health. The presented project was intended as a first step to try to fill this gap, in an attempt to promote and encourage possible control actions.

The goal was to develop and validate a simple, fast and effective method for the analysis of 15 widely used drugs (i.e., benzodiazepines, opioids, antihistaminics, beta-blockers) in whole blood using DBS. The compounds of interest were chosen due to their ototoxic and/or narcoleptic side effects that, indeed, could affect workers’ safety.

The targets were all achieved since the method is low cost, free from any need of pre-treatment, extremely easy to perform and user-friendly. The use of benchtop paper, that is particularly low cost and easily available and avoids any possible interaction with analytes was one of the undeniable benefits. The choice of a triple-quadrupole mass spectrometer and the two MRM transitions for the detection allowed us to achieve a high-sensitivity and an unambiguous identification. The simple ‘dilute and shoot” process is a key factor of the simplicity and cheapness of the method as well as the use of an easy sampling system such as the DBS. The LOQs, achieved applying the entire procedure, were consistent with the therapeutic blood levels of the examined substances.

All these features were crucial in a method intended to encourage its diffusion as a primary prevention intervention, even in small private workplaces. Moreover, unlike the majority of other methods, the presented procedure is able to give a response on 15 substances (from nine different classes) in 36 total minutes from the blood sampling to the results that is a very popular benefit nowadays.

The overall method consumes only a small quantity of dangerous solvents and poses a low biological risk in sample handling. Our Institute, in fact, is at the forefront in safeguarding the health of all workers including the ones working in house, and the presented method is conceived from this perspective. The achieved validation results also proved that the method described herein is suitable for use in different fields such as forensic or clinical analysis.

## Figures and Tables

**Table 1:** Names, structures, adverse effects on the ear and on the nervous system, retention times, optimized MRM transitions (Quantifier and Qualifier), and final MS/MS parameters of the target analytes.

Name	Structure	Ear Disorders	Nervous System Disorders	t_r_ (min)	MRM Transition * (*m*/*z*)	Declustering Potential (V)	Collision Energy (V)	Internal Standard
Atenolol	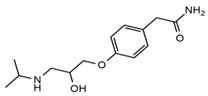		Sleep disturbances, lightheadedness, lethargy, drowsiness, dizziness, vertigo	0.83	267.1/116.1	92	28	Atenolol d_7_
					267.1/73.9		33	
Tramadol	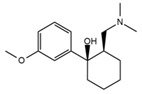		Drowsiness, dizziness, blurred vision	2.23	264.0/58.1	43	43	Tramadol d_6_
					264.0/246.1		17	
Venlafaxine	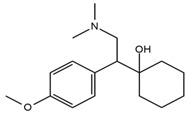	Tinnitus, hyperacusis, otitis media, labyrinthitis	Somnolence, dizziness, tremor, insomnia, blurred vision, vertigo	2.80	278.1/58.0	53	43	
					278.1/260.2		18	
Zolpidem	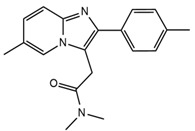	Vertigo, tinnitus, labyrinthitis, otitis externa	Somnolence, attention disorder, hypoesthesia, balance disorder, ataxia, dizziness, blurred vision	3.03	308.0/235.2	82	49	
					308.0/236.1		39	
Triprolidine	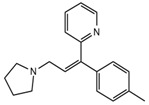		Dizziness, drowsiness, blurred vision	3.28	279.1/208.1	40	22	Diphenhydramine d_3_
					279.1/192.2		63	
Bromazepam	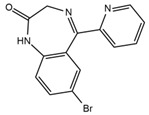		Drowsiness, ataxia, dizziness	3.30	315.9/182.1	82	47	
					315.9/209.1		37	
Trazodone	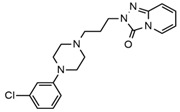	Hypoacusis, tinnitus, vertigo	Dizziness, light-headedness, drowsiness, balance disorder, tremors	3.43	372.1/176.1	82	35	
					372.1/78.0		88	
Fentanyl	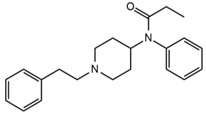		Somnolence, dizziness, confusion, lethargy, tremor, hallucination, insomnia	3.79	337.2/188.1	48	33	
					337.2/105.0		55	
Diphenhydramine	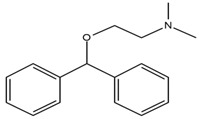	Tinnitus, acute labyrinthitis	Sedation, somnolence, sleepiness, convulsions, tremor, unsteadiness, vertigo, drowsiness, dizziness, attention disorder	3.84	256.1/167.1	35	18	
					256.1/165.1		55	
Escitalopram	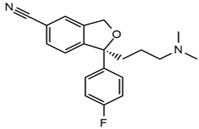	Tinnitus, otitis externa	Somnolence, insomnia, dizziness, lethargy, tremor, amnesia, ataxia	3.93	325.2/262.1	66	29	
					325.2/234.1		39	
Cetirizine	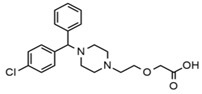	Deafness earache, tinnitus	Somnolence, dizziness, tremor, confusion, leg cramps, paralysis, syncope, vertigo	4.58	389.1/166.1	48	59	Cetirizine d_8_
					389.1/165.1		90	
Cyproheptadine	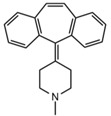	Tinnitus, acute labyrinthitis	Drowsiness, sedation, coordination disorder, loss of coordination, vertigo	4.64	288.0/96.1	94	36	Cyproheptadine d_3_
					288.0/215.1		72	
Alprazolam	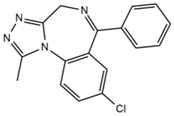	Tinnitus	Ataxia, cognitive dysfunction, blurred vision, attention disorder, confusion, tremor, drowsiness, dizziness	4.67	308.9/205.1	85	55	
					308.9/274.0		36	
Delorazepam	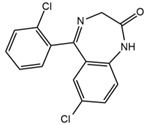		Somnolence, insomnia, dizziness	4.73	305.1/140.0	92	43	
					305.1/242.1		39	
Ebastine	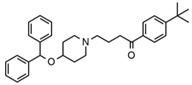		Somnolence, insomnia, dizziness, weakness	6.29	470.1/167.1	65	41	Ebastine d_5_
					470.1/152.1		94	

* For each analyte the first line reported the most intense MRM transition, the quantifier, the second line is for the second most intense, the qualifier.

**Table 2:** Absolute recoveries, method precision (intra and inter-day precision) expressed as relative standard deviation (RSD) and method accuracy expressed as trueness. For each validation level a number of 6 (n = 6) replicates were processed.

Analytes	Absolute Recoveries (R %)			Intra-Day Precision (RSD %)			Inter-Day Precision (RSD %)			Trueness (%)		
	Low (n = 6)	Medium (n = 6)	High (n = 6)	Low (n = 6)	Medium (n = 6)	High (n = 6)	Low (n = 6)	Medium (n = 6)	High (n = 6)	Low (n = 6)	Medium (n = 6)	High (n = 6)
Atenolol	81	85	84	6.1	7.2	9.1	14.2	13.5	14.9	103	91	94
Tramadol	95	91	97	9.8	5.7	10.6	9.8	6.6	6.6	93	92	103
Venlafaxine	75	76	73	8.3	10.0	8.8	10.1	9.6	13.6	78	90	98
Zolpidem	93	89	91	9.1	9.0	7.9	14.4	13.0	13.0	84	98	101
Triprolidine	107	101	100	9.0	10.2	6.2	10.9	6.8	6.8	102	93	100
Bromazepam	76	71	77	9.1	8.6	7.3	14.4	14.0	14.2	86	105	102
Trazodone	93	91	94	4.6	7.6	9.3	8.5	6.9	6.9	103	96	103
Fentanyl	89	95	90	8.1	9.9	6.7	6.3	3.8	3.8	98	101	101
Diphenhydramine	91	91	87	6.8	8.5	10.1	14.7	13.2	13.2	103	88	98
Escitalopram	90	84	88	7.7	10.1	9.6	14.6	14.8	13.8	105	97	98
Cetirizine	75	82	76	9.5	9.7	4.3	7.1	4.3	5.2	99	101	102
Cyproheptadine	85	88	89	6.0	6.7	8.3	9.5	8.8	7.8	105	105	103
Alprazolam	94	99	97	9.7	5.3	9.3	7.3	6.7	6.7	93	100	102
Delorazepam	99	103	106	7.6	2.5	3.4	8.1	6.5	7.2	92	90	101
Ebastine	99	105	100	7.7	7.2	7.8	8.4	6.9	7.3	101	102	98

**Table 3:** Limits of Detection and Quantitation (calculated on the less intense MRM transitions) and matrix effect expressed as decrement of the calibration curve slope. Values of LODs and LOQs were evaluated in whole blood samples as a result of 6 replicates to confirm the value.

Analytes	LODs (pg µL^−1^)	LOQs (pg µL^−1^)	Matrix Effect (%)
Atenolol	0.8	2.4	14
Tramadol	0.1	0.3	13
Venlafaxine	0.7	2.1	9
Zolpidem	0.1	0.3	3
Triprolidine	0.4	1.2	13
Bromazepam	4.9	15	16
Trazodone	0.1	0.3	8
Fentanyl	0.5	1.5	20
Diphenhydramine	0.2	0.6	19
Escitalopram	0.9	2.7	23
Cetirizine	0.2	0.6	13
Cyproheptadine	0.1	0.3	29
Alprazolam	0.5	1.5	8
Delorazepam	0.6	1.8	8
Ebastine	1.4	4.2	16

## Data Availability

The authors declare that the data of this research is available from the corresponding author on request.
